# A good life with psychosis: rate of positive outcomes in first-episode psychosis at 10-year follow-up

**DOI:** 10.1017/S0033291724000205

**Published:** 2024-07

**Authors:** Carmen Simonsen, Gina Åsbø, Mike Slade, Kristin Fjelnseth Wold, Line Widing, Camilla Bärthel Flaaten, Magnus Johan Engen, Siv Hege Lyngstad, Erlend Gardsjord, Thomas Bjella, Kristin Lie Romm, Torill Ueland, Ingrid Melle

**Affiliations:** 1 Early Intervention in Psychosis Advisory Unit for South East Norway, Division of Mental Health and Addiction, Oslo University Hospital, Oslo, Norway; 2 Department of Psychology, University of Oslo, Oslo, Norway; 3 NORMENT, Division of Mental Health and Addiction, Oslo University Hospital and Institute of Clinical Medicine, University of Oslo, Oslo, Norway; 4 School of Health Sciences, Institute of Mental Health, University of Nottingham, Nottingham NG7 2TU, UK; 5 Faculty of Nursing and Health Sciences, Health and Community Participation Division, Nord University, 7801 Namsos, Norway; 6 Nydalen DPS, Division of Mental Health and Addiction, Oslo University Hospital, Oslo, Norway; 7 Section for Treatment of Early Psychosis, Division of Mental Health and Addiction, Oslo University Hospital, Oslo, Norway

**Keywords:** clinical recovery, emotional wellbeing, life satisfaction, positive feelings, personal recovery

## Abstract

**Background:**

More knowledge about positive outcomes for people with first-episode psychosis (FEP) is needed. An FEP 10-year follow-up study investigated the rate of personal recovery, emotional wellbeing, and clinical recovery in the total sample and between psychotic bipolar spectrum disorders (BD) and schizophrenia spectrum disorders (SZ); and how these positive outcomes overlap.

**Methods:**

FEP participants (*n* = 128) were re-assessed with structured clinical interviews at 10-year follow-up. Personal recovery was self-rated with the Questionnaire about the Process of Recovery-15-item scale (total score ⩾45). Emotional wellbeing was self-rated with the Life Satisfaction Scale (score ⩾5) and the Temporal Experience of Pleasure Scale (total score ⩾72). Clinical recovery was clinician-rated symptom-remission and adequate functioning (duration minimum 1 year).

**Results:**

In FEP, rates of personal recovery (50.8%), life satisfaction (60.9%), and pleasure (57.5%) were higher than clinical recovery (33.6%). Despite lower rates of clinical recovery in SZ compared to BD, they had equal rates of personal recovery and emotional wellbeing. Personal recovery overlapped more with emotional wellbeing than with clinical recovery (χ^2^). Each participant was assigned to one of eight possible outcome groups depending on the combination of positive outcomes fulfilled. The eight groups collapsed into three equal-sized main outcome groups: 33.6% clinical recovery with personal recovery and/or emotional wellbeing; 34.4% personal recovery and/or emotional wellbeing only; and 32.0% none.

**Conclusions:**

In FEP, 68% had minimum one positive outcome after 10 years, suggesting a good life with psychosis. This knowledge must be shared to instill hope and underlines that subjective and objective positive outcomes must be assessed and targeted in treatment.

## Introduction

People diagnosed with psychotic disorders are concerned about their chances of recovery and having a good life. They are often presented pessimistic prognoses despite research indicating the potential of positive outcomes (Bressan, Grohs, Matos, & Shergill, 2018). Because hope is important for recovery and a good life, it is crucial to offer balanced information about positive outcomes.

According to the ‘complete state model of mental health’, positive outcomes involve the absence of ‘mental illness’ or the presence of ‘positive mental health’, which are related but distinct unipolar dimensions (Keyes, 2005; Keyes & Martin, 2017; Slade, 2010; Westerhof & Keyes, 2010). A common subdivision of positive outcomes is *clinical recovery* (absence of mental illness), and *personal recovery* or *subjective wellbeing* (presence of positive mental health) (Slade, Oades, & Jarden, 2017). Clinical recovery requires a clinician-rated absence of symptoms combined with a lack of functional impairment for a pre-defined duration. Systematic reviews of first-episode psychosis (FEP) studies report symptomatic remission in over half of participants, with one-third also achieving adequate functioning, thus clinical recovery (Lally et al., 2017; Leonhardt et al., 2017; Van Eck, Burger, Vellinga, Schirmbeck, & de Haan, 2017). In line with traditional perceptions, the FEP 10-year follow-up study we are reporting from here found higher clinical recovery rates for psychotic bipolar spectrum disorders (50%) than for schizophrenia spectrum disorders (23%) (Asbo et al., 2022).

Many people with psychotic disorders report subjective experiences of positive mental health despite continuing clinical symptoms, e.g. being in personal recovery or experiencing subjective wellbeing (Slade, 2010; Slade et al., 2017). The first wave of research into personal recovery involved qualitative studies asking participants what recovery is to them. Several systematic reviews have tried synthesizing this body of research into common themes (Ellison, Belanger, Niles, Evans, & Bauer, 2018; Jagfeld, Lobban, Marshall, & Jones, 2021; Leamy, Bird, Le Boutillier, Williams, & Slade, 2011; Stuart, Tansey, & Quayle, 2017; Wood & Alsawy, 2018). The conceptual framework by Leamy et al. (2011), arriving at five personal recovery processes in mental illness; *connectedness, hope, identity, meaning* and *empowerment,* abbreviated to CHIME, is widely recommended and used in research (Leendertse et al., 2021).

Despite agreement that personal recovery is a continuous process, the existing conceptual frameworks describing personal recovery have inspired the development of several self-rating scales allowing the quantitative investigation of personal recovery as an outcome (Law, Morrison, Byrne, & Hodson, 2012; Shanks et al., 2013; Sklar, Groessl, O'Connell, Davidson, & Aarons, 2013). Two systematic reviews favored the Recovery Assessment Scale (RAS) (Corrigan, Salzer, Ralph, Sangster, & Keck, 2004) and the Questionnaire about the Process of Recovery (QPR) (Neil et al., 2009) due to good psychometric properties (Shanks et al., 2013; Sklar et al., 2013). The RAS has since been most frequently used (Leonhardt et al., 2017), yet, the QPR has been preferred in psychosis research because it was developed specifically for this target group in collaboration with service users (Best, Law, Pyle, & Morrison, 2020) and aligns well with the five CHIME processes (Lim, Li, Xie, Tan, & Lee, 2019; Shanks et al., 2013).

The few FEP studies that have investigated the rate of personal recovery report 46.9% at 1-year follow-up (Dubreucq et al., 2022), 35% at 2-year follow-up (Austin, Hjorthoj, Baagland, Simonsen, & Dam, 2022), and 53.7% and 51.9% at 20-year follow-up (O'Keefe et al., 2019; Peralta et al., 2022). Lower rates were found for people with psychosis that were in need of treatment, with 14.5% in a cognitive-behavioral therapy study (Best et al., 2020) and 17.4% in an integrative family intervention study (Yu et al., 2022). Moreover, research suggests that in line with clinical recovery, personal recovery may be higher in psychotic bipolar compared to schizophrenia spectrum disorders (Asbo et al., 2022; Vass, Sitko, West, & Bentall, 2017), but this requires further research.

A useful conceptualization of subjective wellbeing is a three-way subdivision into *social wellbeing* (relationships; community), *psychological wellbeing* (meaning in life; autonomy), and *emotional wellbeing* (life satisfaction; positive feelings) (Austin, 2018; Keyes & Martin, 2017; Westerhof & Keyes, 2010). Studies of participants with psychotic disorders report both average and lower levels of subjective (Chan, Mak, Chio, & Tong, 2017; Stanga, Turrina, Valsecchi, Sacchetti, & Vita, 2019) and emotional wellbeing (Gardsjord et al., 2016; Mankiewicz, Gresswell, & Turner, 2013a, 2013b; Melle et al., 2005; Palmer, Martin, Depp, Glorioso, & Jeste, 2014; Saperia et al., 2018; Tso, Grove, & Taylor, 2014; Visser, Chapman, Ruiz, Raugh, & Strauss, 2020). However, there are few investigations of the rate of good subjective wellbeing in FEP, and the potential differences between people with psychotic bipolar and schizophrenia spectrum disorders (Stanga et al., 2019; Tso et al., 2014), requiring further research.

A close relationship between personal recovery and subjective wellbeing was supported by a prospective schizophrenia study reporting that subjective wellbeing (using the above three-way conception) was predicted more by personal recovery than by clinical recovery (Chan et al., 2017). Moreover, a conceptual framework of wellbeing in psychotic disorders identified seven wellbeing indicators comprising: *positive feelings*, *symptom relief*, *connectedness*, *hope*, *self-worth*, *meaning*, and *empowerment* (Schrank et al., 2014). The last five wellbeing indicators mirror the five personal recovery CHIME processes as well as social and psychological wellbeing, illustrating their conceptual overlap. However, how CHIME processes (personal recovery) relate to *positive feelings* (emotional wellbeing) or *symptom relief* (clinical recovery) remains less obvious and should be investigated in FEP.

Several recent systematic reviews and individual studies report associations with small effect sizes between personal recovery and clinical recovery in terms of psychotic symptoms (positive and negative) and functioning, while, the associations between personal recovery and affective symptoms have medium effect sizes (Leendertse et al., 2021; Leonhardt et al., 2017; Van Eck et al., 2017; Yu et al., 2022). Thus, it is generally agreed that despite the overlap between personal and clinical recovery they are distinct constructs (Leendertse et al., 2021; van Weeghel, van Zelst, Boertien, & Hasson-Ohayon, 2019), in line with the complete state model (Keyes, 2005). This requires further research in FEP.

The aim of the present study is to investigate the prevalence of positive outcomes in people with psychotic disorders. To fill the above knowledge gaps, we explored two research questions in a 10-year follow-up of participants with FEP: (1) What is the rate of *personal recovery*, *emotional wellbeing*, and *clinical recovery* in the total sample and between bipolar and schizophrenia spectrum disorders? (2) What is the overlap between *personal recovery*, *emotional wellbeing*, and *clinical recovery* in the total sample?

## Methods

### Participants

From 2004 to 2012, 444 participants with FEP were recruited to the Thematically Organized Psychosis (TOP) study at the Norwegian Centre for Mental Disorders Research (NORMENT) from most in- and outpatient psychiatric services in the Oslo area, Norway. From 2015 to 2021, they were invited to participate in a 10-year follow-up, with 169 participants being re-assessed. *Inclusion criteria* at baseline and 10-year follow-up involved fulfilling the criteria for a psychotic disorder; i.e. a DSM-IV diagnosis of *broad schizophrenia spectrum disorder* (schizophrenia, schizophreniform disorder, schizoaffective disorder, and ‘other psychoses’ [delusional disorder, brief psychotic disorder, and psychotic disorder not otherwise specified]) or *psychotic bipolar spectrum disorder* (bipolar I disorder, bipolar II disorder, and bipolar disorder not otherwise specified with a history of psychotic symptoms), and the ability to give informed consent. Inclusion criteria at baseline also included less than 1 year since the start of first adequate treatment for one of the above diagnoses, age of 18–65 years, and adequate Scandinavian language skills. In the present study, the 10-year follow-up diagnosis was used to form the two diagnostic groups; *schizophrenia spectrum disorder* and *psychotic bipolar spectrum disorder*, hereafter called ‘schizophrenia group’ and ‘bipolar group’. *Exclusion criteria* were previous brain injury requiring hospitalization, neurological, or other medical condition causing psychotic symptoms.

**Figure 1.**
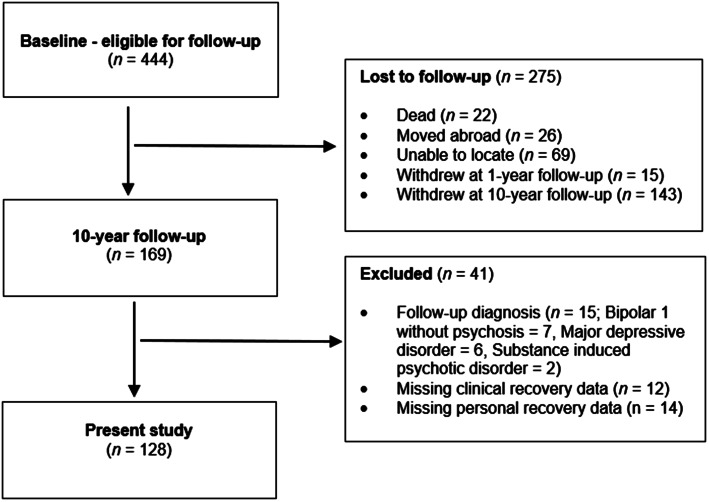
Participation in 10-year follow-up.

Figure 1 provides a flowchart describing participation in the present study. Amongst the 444 baseline FEP participants, 169 completed the 10-year follow-up assessment, resulting in a 38.1% retention rate. After assessment at 10-year follow-up, 15 participants were excluded due to a revised diagnosis outside the two diagnostic groups described above, and 12 participants because of insufficient information to determine clinical recovery, resulting in 142 participants described in our previous study on clinical recovery (Asbo et al., 2022). The number of participants in the present study was further reduced to 128 because 14 participants had not completed the personal recovery measure (QPR-15).

The only significant baseline demographic and clinical difference between completers and non-completers of the 10-year follow-up was more men (63.6% *v*. 52.7%) (χ^2^ [1] = 5.229, *p* < 0.05) and schizophrenia diagnoses (66.6% *v.* 60.9%) (χ^2^ [1] = 9.110, *p* < 0.05) in the non-completion group.

The Regional Committee for Medical Research Ethics and the Norwegian Data Inspectorate approved the study. Written informed consent to participation and data was collected in compliance with the regulations of our institutions. Study methodology was pre-registered in the Open Science Framework: https://osf.io/sjrkv/

### Assessment and measures

#### Demographic and clinical characteristics

*Demographic and clinical data* were collected from comprehensive clinical interviews and medical charts. At baseline and 10-year follow-up the same well-known, frequently used, valid, and reliable clinical measures and procedures were used. Assessment and diagnosis were carried out and rated by licensed clinical psychologists or physicians with psychiatric training supervised by experienced consultant psychiatrists and clinical psychologists and were discussed until agreement by consensus rating in weekly meetings. *Diagnoses* was based on the Structural Clinical Interview for DSM-IV Axis I disorders (SCID-I) (First, Spitzer, Gibbon, & Williams, 1995). *Psychosis* was initially assessed with the Positive and Negative Syndrome Scale (PANSS) (Kay, Fiszbein, & Opler, 1987). *Positive*, *negative*, *disorganized*, *excited*, *and depressive symptoms* were measured with Wallwork's five-factor model of PANSS (Wallwork, Fortgang, Hashimoto, Weinberger, & Dickinson, 2012) found to be appropriate for FEP populations (Langeveld et al., 2013). *Depressive symptoms* were measured further with both The Calgary Depression Scale for Schizophrenia (CDSS) (Addington, Addington, & Schissel, 1990) and Inventory of Depressive Symptoms – Clinician rated (IDS-C) (Rush, Gullion, Basco, Jarrett, & Trivedi, 1996), developed for schizophrenia and affective disorders respectively. *Manic symptoms* were measured with the Young Mania Rating Scale (YMRS) (Young, Biggs, Ziegler, & Meyer, 1978). *Global functioning* was measured with the Global Assessment of Functioning Scale (GAF) (Pedersen, Hagtvet, & Karterud, 2007), split version. *Alcohol and drug use* were measured with the Alcohol Use Disorder Identification Test and Drug Use Disorders Identification Test (AUDIT/DUDIT) (Berman, Bergman, Palmstierna, & Schlyter, 2005; Saunders, Aasland, Babor, de la Fuente, & Grant, 1993).

#### Positive outcomes

*Personal recovery* was measured with the self-rated Questionnaire about the Process of Recovery 15-item version (QPR-15) (Law, Neil, Dunn, & Morrison, 2014), because it was originally developed in collaboration with service users specifically for people with psychosis (Neil et al., 2009), with all items matching CHIME processes (Lim et al., 2019; Shanks et al., 2013). The 15-item version was used rather than the original 22-item version because of better psychometric properties (Law et al., 2014; Williams et al., 2015); and later validation for bipolar disorder (Kraiss et al., 2019). Agreement with 15 statements like ‘*I feel that my life has a purpose*’ are rated 0–4 (‘strongly disagree’ to ‘strongly agree’). Total scores range from 0 to 60, with personal recovery defined as QPR-15 total score ⩾45 (mean score minimum 3 ‘agree’) (Best et al., 2020).

*Emotional wellbeing* comprises life satisfaction and positive feelings (experience of pleasure).

*Life satisfaction* was measured with the global self-rated ‘Satisfaction with life in general’ item in the Lehman Quality of Life Interview, brief version (Lehman, 1988; Melle et al., 2005). This item, hereafter called Life Satisfaction Scale (LSS), is rated 1–7 (‘very dissatisfied’ to ‘very satisfied’). Good life satisfaction was defined as an LSS score ⩾5 (‘slightly satisfied’ or better).

*Positive feelings* (*experience of pleasure*) were measured with the self-rated Temporal Experience of Pleasure Scale (TEPS) (Gard, Gard, Kring, & John, 2006), designed to investigate anticipatory and consummatory pleasure in schizophrenia spectrum disorders. We used the scale as a general measure of *pleasure*, functioning as a proxy measure of positive feelings. Eighteen statements like ‘*I appreciate the beauty of a fresh snow fall*’ is rated 1–6 (‘very false for me’ to ‘very true for me’). Item number 13 was reversed as required (Gard et al., 2006). Total scores range from 18 to 108, with experience of pleasure defined as a TEPS total score ⩾72 (mean score minimum 4 ‘slightly true for me’).

*Clinical recovery* was defined according to recent suggestions from our research group which are suitable for affective and non-affective psychosis (Asbo et al., 2022). The criteria involved: (1) *symptom remission* consisting of: *psychotic symptom remission* (according to the international consensus definition with PANSS scores P1/P2/P3/G5/G9/N1/N4/N6 below 4 [Andreasen et al., 2005]) and *affective symptom remission* (IDS-C score below 14, CDSS-score below 7 and YMRS-score below 8, as well as not meeting criteria for a current affective episode according to SCID-1 at follow-up); (2) *adequate functioning* consisting of: *occupational functioning* (part-time [⩾40%]), *social functioning* (having a close friend/confidant), and *independent living* (residing in unsupervised home and maintaining activities of daily living); (3) *duration* of symptom remission and adequate functioning for a minimum of 12 months.

### Statistical analysis

Analyses were completed with IBM SPSS Statistics version 26. The differences in background characteristics between 10-year follow-up completers *v.* non-completers and between schizophrenia *v.* bipolar diagnostic groups were investigated with Chi-square, *t* test or Mann–Whitney *U* test dependent on the data in question. First, mean total scores and rates of participants fulfilling the criteria for personal recovery, good emotional wellbeing, and clinical recovery in the total sample were investigated, followed by the difference between schizophrenia and bipolar groups using *t* tests and Chi-square. Second, the overlap between personal recovery and emotional wellbeing and clinical recovery, respectively in the total sample, was investigated using Pearson correlations and Chi-square with strengths based on Cohen's criteria (Cohen, 1988). All tests were two-tailed. To reduce the chance of type 1 errors due to multiple testing, Bonferroni correction was carried out by dividing the preset significance level with number of outcomes (0.05/8) = 0.006. The overlap was visually illustrated in a Venn diagram developed by assigning each participant to one of eight possible outcome groups depending on the combination of positive outcome criteria they fulfilled ([1] personal recovery/emotional wellbeing/clinical recovery; [2] personal recovery/clinical recovery; [3] personal recovery; [4] personal recovery/emotional wellbeing; [5] emotional wellbeing; [6] emotional wellbeing/clinical recovery; [7] clinical recovery; [8] none).

## Results

### Background characteristics

Table 1 presents 10-year follow-up demographic and clinical characteristics for the total sample, schizophrenia group, and bipolar group. Significant demographic and clinical differences between the two diagnostic groups were less education, lower GAF-F, and higher PANSS-positive, negative, disorganized, and manic symptom scores in the schizophrenia group.

**Table 1.** Demographic and clinical characteristics at 10-year follow-up in total sample, and between schizophrenia group and bipolar group

		Total sample (*n* = 128)	Schizophrenia spectrum (*n* = 88)	Bipolar spectrum (*n* = 40)	Chi-square/ *t* test/Mann–Whitney U		
					χ^2^/*t*/*u*	df	*p*
**Demographics**							
Gender, female	*n* (*%*)	61 (47.7)	38 (43.2)	23 (57.5)	χ^2^ = 1.72	1	0.189
Age	*M* (s.d.)	37.6 (8.1)	37.1 (8.0)	38.8 (8.4)	*t* = −1.05	126	0.296
Non-Norw. country of origin^a^	*n* (*%*)	24 (18.8)	16 (21.1)	8 (22.2)	χ^2^ = 0.00	1	1.000
In current relationship	*n* (*%*)	57 (44.5)	35 (39.8)	22 (55.0)	χ*^2^* = 2.00	1	0.157
Education, years	*M* (s.d.)	13.6 (2.5)	13.0 (2.5)	14.8 (2.0)	*t* = −3.92	126	**<0.001**
**Diagnosis**							
*Schizophrenia spectrum*							
Schizophrenia	*n* (*%*)	50 (39.1)	50 (56.8)	-			
Schizophreniform	*n* (*%*)	1 (0.8)	1 (1.1)	-			
Schizoaffective	*n* (*%*)	14 (10,9)	14 (15.9)	-			
‘Other psychoses’ ^b^	*n* (*%*)	23 (18.0)	23 (26.1)	-			
*Bipolar spectrum*							
Bipolar 1 disorder	*n* (*%*)	36 (28.1)	-	36 (90.0)			
Bipolar 2 disorder	*n* (*%*)	3 (2.3)	-	3 (7.5)			
Bipolar NOS	*n* (*%*)	1 (0.8)	-	1 (2.5)			
**Number of episodes**^c^							
Psychotic	*Mdn* (*ran*)	2 (0–16)	2 (0-10)	2 (0-16)	*U* = 1991.0	126	0.168
Manic	*Mdn* (*ran*)	1 (0–9)	1 (0-4)	2 (0-9)	*U* = 554.5	126	0.**013**
Depressive	*Mdn* (*ran*)	2 (0–48)	2 (0-32)	3 (0-48)	*U* = 1256.5	126	0.341
**Clinical symptoms**^d^							
PANSS positive	*M* (s.d.)	7.3 (4.0)	8.2 (4.4)	5.2 (2.0)	*t* = 5.29	126	**<0.001**
PANSS negative	*M* (s.d.)	9.2 (4.8)	9.8 (5.4)	7.8 (2.8)	*t* = 2.84	126	**0.005**
PANSS disorganized	*M* (s.d.)	4.2 (2.0)	4.5 (2.3)	3.7 (0.9)	*t* = 2.77	126	**0.007**
PANSS excited	*M* (s.d.)	4.7 (1.5)	4.8 (1.6)	4.5 (1.0)	*t* = 1.40	126	0.165
PANSS depressive	*M* (s.d.)	6.4 (3.3)	6.6 (3.3)	6.6 (3.3)	*t* = 1.09	126	0.276
CDSS	*M* (s.d.)	3.2 (4.4)	3.4 (4.6)	2.8 (4.1)	*t* = 0.71	126	0.481
YMRS	*M* (s.d.)	3.3 (4.1)	3.9 (4.4)	1.9 (2.6)	*t* = 3.20	126	0*.***002**
**Global functioning**^d^							
GAF-F	*M* (s.d.)	60.8 (17.2)	56.7 (16.0)	70.0 (16.3)	*t* = −4.34	126	**<0.001**
**Substance use**^d^							
AUDIT	*Mdn* (*ran*)	3 (0–23)	4 (0-23)	3 (0-20)	*U* = 1320.0	126	0.803
DUDIT	*Mdn* (*ran*)	0 (0–35)	0 (0-35)	0 (0-23)	*U* = 1124.5	126	0.068
**Medication**^e^							
Antipsychotics	*n* (*%*)	85 (67.5)	58 (67.4)	27 (67.5)	χ*^2^* = 0.00	1	1.000

*Note: n* (%) = number and percentage, M (SD) = mean (standard deviation), Mdn (ran) = median (range). Bold numerals indicate statistically significant difference.

a Non-Norwegian country of origin *n* = 112.

b Other psychoses: delusional disorder = 5; brief psychotic disorder = 1; psychotic disorder not otherwise specified = 19.

c Number of episodes during 10-year follow-up period.

d Positive and Negative Symptom Scale (PANSS); Calgary Depression Scale for Schizophrenia (CDSS); Young Mania Rating Scale (YMRS); Global Assessment of Functioning-Functioning subscale (GAF-F); Alcohol Use Disorders Identification Test (AUDIT); Drug Use Disorder Identification Test (DUDIT).

e Medication *n* = 126.

### Rates of positive outcomes: personal recovery, emotional wellbeing, and clinical recovery

Table 2 presents mean total scores and the percentage of participants fulfilling the criteria and definition of personal recovery, emotional wellbeing, and clinical recovery in the total sample. The mean QPR-15 total score was close to the cut-off for *personal recovery* (⩾45), with over half the participants meeting the criteria of personal recovery (50.8%). Mean LSS score was at the cut-off for good *life satisfaction* (score⩾5), with over half the participants meeting the criteria of good life satisfaction (60.9%). Mean TEPS total score was almost above the cut-off for *experience of pleasure* (score⩾72), with over half the participants meeting the criteria of experiencing pleasure (57.9%). As reported in our previous paper involving an overlapping sample (Asbo et al., 2022), approximately one-third fulfilled the criteria for *clinical recovery* (33.6%). Finally, almost one-third had no positive outcomes (32.0%).

**Table 2.** Rates of positive outcomes at 10-year follow-up in total sample, and between schizophrenia group and bipolar group

		Total sample (*n* = 128)	Schizophrenia spectrum (*n* = 88)	Bipolar spectrum (*n* = 40)	*t* test/Chi-square		
					*t*/χ^2^	df	*p*
**Personal recovery**							
*Personal recovery* (*QPR-15*)^a^							
Total score (1–60)	*M* (s.d.)	43.6 (11.4)	43.1 (11.1)	44.6 (12.1)	*t* = −0.70	126	0.483
*Yes* (total score ⩾45)	*n* (*%*)	65 (50.8)	41 (46.6)	24 (60.0)	χ*^2^* = 1.48	1	0.224
**Emotional wellbeing**							
*Life satisfaction* (*LSS*)^b^							
Score (1–7)	*M* (s.d.)	5.0 (1.4)	4.9 (1.5)	5.2 (1.3)	*t* = −1.29	126	0.199
*Yes* (score ⩾5)	*n* (*%*)	78 (60.9)	52 (59.1)	26 (65.0)	χ*^2^* = 0.19	1	0.660
*Pleasure* (*TEPS*)^c^							
Total score (18–108)	*M* (s.d.)	71.5 (15.7)	70.5 (14.7)	73.8 (17.9)	*t* = −1.05	112	0.295
*Yes* (total score ⩾72)	*n* (*%*)	66 (57.9)	40 (50.6)	26 (74.3)	χ*^2^* = 4.64	1	*0.031*
**Clinical recovery**^d^							
*Yes* (Asbo et al., 2022)	*n* (*%*)	43 (33.6)	20 (22.7)	23 (57.5)	χ*^2^* = 13.39	1	**<0.001**
**No positive outcomes**	*n* (*%*)	41 (32.0)	31 (35.2)	10 (25.0)	χ*^2^* = .89	1	0.345

*Note: n* (%) = number and percentage, M (s.d.) = mean (standard deviation).

Bold numerals indicate statistically significant difference after Bonferroni correction (significance level = 0.006). Italics numerals indicate no longer statistically different after Bonferroni correction.

a Personal recovery = Questionnaire about the Process of Recovery – 15-item (QPR-15): total score ⩾45.

b Life satisfaction = Life Satisfaction Scale (LSS): score ⩾5.

c Pleasure (positive feelings) = Temporal Experience of Pleasure Scale (TEPS): total score ⩾72 (*n* = 114).

d Clinical recovery = symptomatic remission and adequate functioning for minimum 12 months (criteria from Asbo et al., 2022).

Table 2 also presents mean total scores and the rates meeting the criteria of personal recovery, emotional wellbeing, and clinical recovery, as well as rates with no positive outcomes, between the schizophrenia and bipolar groups. In both groups, the mean total scores for QPR-15, LSS, and TEPS were close to the cut-off for personal recovery, life satisfaction, and experienced pleasure, respectively. There were no significant differences in outcome between the two diagnostic groups, apart from significantly lower rate of clinical recovery in the schizophrenia group compared to the bipolar group (as reported in our previous paper [Asbo et al., 2022]).

### Overlap between positive outcomes: personal recovery, emotional wellbeing, and clinical recovery:

In the total sample, the total score of QPR-15 had a strong correlation (Pearson [Cohen, 1988] with the total scores of: LSS [*r* = 0.793, *p* < 0.001] and TEPS [*r* = 0.607, *p* < 0.001]).

Table 3 presents the difference in emotional wellbeing and clinical recovery between participants with and without personal recovery (χ^2^). A significantly higher percentage of participants with personal recovery met the criteria for life satisfaction (93.8%), experienced pleasure (66.7%), and clinical recovery (52.3%) compared to participants without personal recovery. The association between personal recovery and life satisfaction had a strong effect size, while the association between personal recovery and experience of pleasure and clinical recovery had a moderate effect size (Cohen, 1988). Thus, personal recovery overlapped more strongly with life satisfaction, than with experience of pleasure and clinical recovery.

**Table 3.** Overlap between positive outcomes at 10-year follow-up in total sample: emotional wellbeing and clinical recovery in participants with and without personal recovery

		Personal recovery - *No* (*n* = 63)	Personal recovery - *Yes* (*n* = 65)	Chi-square			
				χ^2^	df	*p*	*phi*
**Emotional wellbeing**							
*Life satisfaction* (*LSS*)							
*Yes* (score ⩾5)	*n* (*%*)	17 (27.0)	61 (93.8)	χ^2^ *=* 57.31	1	**<0.001**	0.685
*Pleasure* (*TEPS*)							
*Yes* (total score ⩾72)	*n* (*%*)	22 (33.3)	44 (66.7)	χ^2^ *=* 14.17	1	**<0.001**	0.370
**Clinical recovery**							
*Yes* (Asbo *et al*., 2022)	*n* (*%*)	9 (14.3)	34 (52.3)	χ^2^ *=* 19.06	1	**<0.001**	0.402

*Note: n* (%) = number and percentage.

Bold numerals indicate statistically significant difference.

Personal recovery = Questionnaire about the Process of Recovery, 15-item (QPR-15): ⩾45; Life satisfaction = Life Satisfaction scale (LSS): score ⩾5; Pleasure = Temporal Experience of Pleasure Scale (TEPS): total score ⩾72) (*n* = 114); Clinical recovery = symptomatic remission and adequate functioning for minimum 12 months (criteria from Asbo et al., 2022).

**Figure 2.**
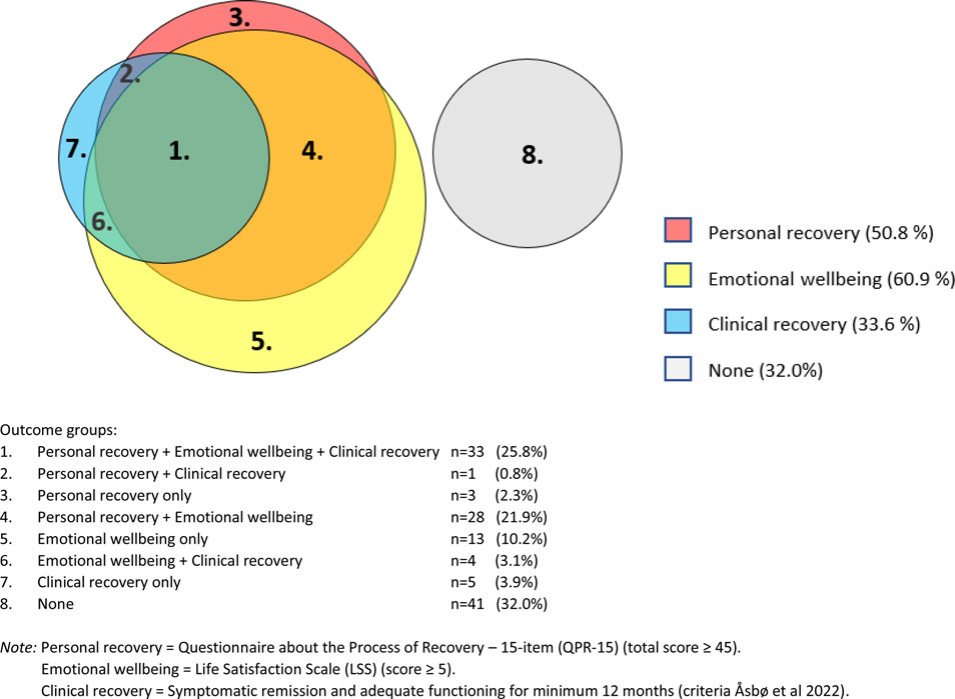
Overlap between positive outcomes at 10-year follow-up in the total sample (*n* = 128): each participant assigned to one of eight groups depending on combination of positive outcomes.

Figure 2 is a Venn diagram visually illustrating the overlap between positive outcomes in the total sample by assigning each participant to one of eight outcome groups depending on their combination of positive outcomes. Firstly, the figure illustrates that the percentage fulfilling criteria for personal recovery (groups 1, 2, 3, 4) and emotional wellbeing (groups 1, 4, 5, 6) is higher than for clinical recovery (groups 1, 2, 6, 7). Secondly, Fig. 2 illustrates that personal recovery overlaps more with emotional wellbeing in terms of life satisfaction (groups 1, 4) than with clinical recovery (groups 1, 2). Hence, amongst the 64.1% (*n* = 82) experiencing personal recovery and/or emotional wellbeing, the majority experienced both (groups 1, 4); approximately half did not additionally experience clinical recovery (groups 3, 4, 5), while the other half did (groups 1, 2, 6). Moreover, amongst the 33.6% (*n* = 43) that were in clinical recovery, the majority also experienced personal recovery and/or emotional wellbeing (groups 1, 2, 6), while a minority did not (group 7). Thirdly, Fig. 2 illustrates that the eight outcome groups collapse into three equally sized main outcome groups: (1) the 33.6% rated clinically recovered (groups 1, 2, 6, 7) generally also experienced personal recovery and/or emotional wellbeing; (2) another 34.4% experienced personal recovery and/or emotional wellbeing without clinical recovery (groups 3, 4, 5); (3) lastly, 32% had no positive outcomes (group 8). Finally, Fig. 2 illustrates that 68% (*n* = 87) of the participants had at least one positive outcome.

## Discussion

The contribution of this FEP 10-year follow-up study is twofold. Firstly, rates of personal recovery (50.8%) and emotional wellbeing (life satisfaction [60.9%] and pleasure [57.9%]) were higher than rates of clinical recovery (33.6%). Despite lower rates of clinical recovery in the schizophrenia group compared to the bipolar group, their rates of personal recovery and emotional wellbeing, as well as having no positive outcomes were equal. Secondly, personal recovery overlapped strongly with emotional wellbeing and moderately with clinical recovery. This resulted in three equally sized outcome groups: one-third experienced personal recovery and/or emotional wellbeing *and* clinical recovery; one-third experienced personal recovery and/or emotional wellbeing *without* clinical recovery; one-third had no positive outcomes. In sum, 68% experienced at least one positive outcome, while 25.8% had all three positive outcomes.

The rate of personal recovery of 50.8% in the present 10-year follow-up study is relatively consistent with the four previous FEP studies reporting 46.9% at 1-year follow-up, 35% at 2-year follow-up, 53.7% and 51.9% at 20-year follow-up (Austin et al., 2022; Dubreucq et al., 2022; O'Keefe et al., 2019; Peralta et al., 2022), of which two also used the QPR-15. Thus, the proportion of FEP participants experiencing personal recovery seems to be fairly consistent across the first 20 years after start of treatment. The present study additionally investigated emotional wellbeing. Our finding of high levels and rates of life satisfaction is in line with an FEP study reporting life satisfaction in the ‘fair’ to ‘good’ range at 5-year follow-up (Melle et al., 2005), with improvements at 10-year follow-up (Gardsjord et al., 2016). Our high level and rate of experienced pleasure corroborates previous studies reporting only slightly lower levels of experienced pleasure in schizophrenia compared to healthy controls (Visser et al., 2020), and equal levels of positive emotions across people with schizophrenia and healthy controls (Mankiewicz, Gresswell, & Turner, 2013b; Saperia et al., 2018). Finally, the present rate of clinical recovery mimics the latest meta-analysis of clinical recovery in FEP (38%) (Lally et al., 2017).

The present higher rate of personal recovery and emotional wellbeing compared to clinical recovery could be explained by differences in *content* and *duration criteria* across these constructs. Compared to personal recovery and emotional wellbeing, clinical recovery reflects an absence of mental illness rather than the presence of positive mental health. Moreover, clinical recovery involves a minimum 1-year duration rather than the less specific and potentially shorter timeframes for the other two constructs (‘generally/lately’ and ‘specifically the last week’). Previously, we found that with the absence of a duration criteria, clinical recovery rate increased from 31.7% to 36.6% (Asbo et al., 2022). Further research investigating the individual impact of these factors on rates of personal recovery and emotional wellbeing is required.

To the best of our knowledge, we are among the first to report equally high levels of self-rated personal recovery and emotional wellbeing in schizophrenia and psychotic bipolar groups, despite less education, lower functioning, and more psychotic symptoms in the schizophrenia group. This does not align with a previous study reporting that the experience of pleasure was higher in bipolar disorders than in schizophrenia disorders despite equal levels of negative affect and trait anhedonia (Tso et al., 2014). This inconsistency calls for further research. Moreover, whether lack of experiencing pleasure (anhedonia) and problems with anticipated rather than the consummatory experience of pleasure are displayed by people with psychotic bipolar disorder needs investigation. Nevertheless, our findings suggest that although people with schizophrenia disorders have lower chances of clinical recovery, they have equal chances of experiencing positive mental health in terms of experiencing a connected, hopeful, meaningful, empowered, and satisfying life, including pleasure.

The findings related to our second research question revealed that personal recovery overlaps more with emotional wellbeing than with clinical recovery, consistent with the complete state model of mental health (Keyes, 2005). The limited overlap between personal recovery and clinical recovery is consistent with several recent systematic reviews concluding that personal and clinical recovery remain distinct constructs (Leendertse et al., 2021; Leonhardt et al., 2017; Van Eck et al., 2017; van Weeghel et al., 2019). Nevertheless, our understanding of these recovery constructs and their relationship may be improved by a promising project currently exploring convergent and concurrent validity between them (Pelletier et al., 2020), as well as investigations of other related factors such as attachment style (van Bussel et al., 2021). The overlap we found between personal recovery and emotional wellbeing rather than clinical recovery is in line with the robust findings that personal recovery predicts subjective wellbeing above and beyond that of clinical recovery (Chan et al., 2017). These relationships are likely not only explained by personal recovery and emotional wellbeing being more similar in terms of content and duration criteria, but also because they are both self-rated, rather than clinician-rated.

Among the three equally sized main outcome groups, the first (33.6%) fulfilled criteria for clinical recovery, with nearly all participants also fulfilling criteria for personal recovery and/or emotional wellbeing. Thus, the absence of mental illness without the presence of positive mental health is rare in psychotic disorders. This is the best pattern of outcome called *complete mental health* or *flourishing* according to the complete state model (Slade, 2010). A second main outcome group (34.4%) fulfilled criteria for personal recovery and/or emotional wellbeing, but not clinical recovery. Thus, the presence of positive mental health does not depend on absence of mental illness, which is in line with the complete state model of mental health (Keyes, 2005). The third main outcome group (32.0%) had neither absence of mental illness nor presence of positive mental health, which is clearly the poorest pattern of outcome.

Our findings have clinical implications. They provide evidence for personal recovery and emotional wellbeing in two-thirds of participants with FEP after 10 years, of which half were also rated clinically recovered. This demonstrates that presence of positive mental health is possible despite remaining signs of mental illness. Although the absence of mental illness was less common in schizophrenia than in bipolar disorders, the presence of positive mental health was equally common across the two diagnostic groups. Firstly, sharing this evidence with people with psychotic disorders (and their caregivers) is crucial as it may increase their hope and optimism for the future, reduce internalized stigma, which in turn may improve their recovery and chances for a good life. These findings should also be shared with health professionals and students in clinical education as evidence for therapeutic pessimism being empirically wrong. Secondly, these findings suggest that personal recovery and emotional wellbeing are important components of clinical assessment, and that in line with the promotion of patient-reported outcome measures, we need subjective measures of outcome in treatment settings to sufficiently monitor the effectiveness of interventions. Finally, these findings support that positive mental health promotion should be a treatment target alongside reducing symptoms of mental illness, with positive psychotherapy for psychosis (Chu et al., 2022; Schrank et al., 2016) being one possible specific intervention. This might prove to be especially fruitful for the one-third with no positive outcomes.

The main strength of this study is the 10-year investigation of self-rated positive outcome rates in a large sample of FEP, recruited in a country with a public mental health-care system using catchment-area patient admittance, including both psychotic bipolar and schizophrenia spectrum disorders. The clinical assessment was thorough, and the scales used to measure personal recovery, emotional wellbeing, and clinical recovery were carefully chosen because of their psychometric properties and validation in the investigated diagnostic groups. An obvious limitation is the low retention rate (38.1%), possibly caused by extensive baseline and 1-year assessments, and that it is harder to locate and contact participants in more recent longitudinal studies due to increased mobility and changes in privacy legislation (Homman, Smart, O'Neill, & MacCabe, 2021). Participants were also unable to complete assessment because of very poor outcome (too symptomatic) or very good outcome (too busy with work). Nevertheless, we found no baseline differences between the 10-year follow-up completers and non-completers, except for more men and schizophrenia spectrum disorders in the latter group, which should have limited impact as rates of positive mental health did not differ across gender or diagnostic group.

In conclusion, this study shows that most people with FEP have positive mental health, with and without the absence of mental illness, 10 years after they started treatment. This provides evidence for the potential of a good life with psychosis. These findings should be shared with people with psychosis, their caregivers, health professionals, students in clinical education, and the society at large, in order to improve hope and optimism and reduce stigma. This may increase the chances of recovery and a good life. The findings also imply that treatment targets should involve increasing positive mental health alongside reducing mental illness, and that subjective and not only objective measures of positive outcomes are required to adequately monitor the effectiveness of interventions. Finally, we encourage future research exploring positive mental health outcomes in FEP and factors linked to the interrelatedness of positive outcomes to enhance our understanding of these phenomena.
